# Herbal Medicine after Interventional Therapy in Cardiovascular Diseases: Efficacy, Mechanisms, and Safety

**DOI:** 10.1155/2015/603701

**Published:** 2015-11-04

**Authors:** Dazhuo Shi, Michael Y. H. Shen, Honglin Luo, Yan Ma

**Affiliations:** ^1^Department of Cardiology, Xiyuan Hospital of China Academy of Chinese Medicine Sciences, Beijing, China; ^2^Department of Cardiovascular Medicine, Cleveland Clinic Florida, Fort Lauderdale, Weston, FL, USA; ^3^Department of Pathology & Laboratory Medicine, University of British Columbia, Vancouver, BC, Canada; ^4^Medizinische Universitat Wien, Vienna, Austria

Despite recent medical advances, cardiovascular diseases (CVDs) remain the predominant cause of morbidity and mortality all over the world. Interventional therapy (IT) is the milestone of therapy of CVDs and has developed rapidly in recent years. Despite its proven benefit, recurrent cardiovascular events are still a big challenge in cardiology field. Plants have been used for medicinal purposes for as long as history has been recorded. Great varieties of plants are used for medicinal treatments and many new drugs have been discovered from herbal sources. Herbal medicine (HM) means use of natural plant substances (botanicals) to treat and prevent illness. Based on the integrative medicine of eastern and western, the application of HM has valuable significance in reducing the risk of cardiovascular event. During the past decades, some HM products went into Europe and United States for prevention and treatment of CVDs or prevention of in-stent restenosis after IT, however, as the complementary and alternative remedies. Widespread use has increased much demands that HM be regulated as drugs to insure the efficacy, mechanisms, and safety.

This special issue is a collection of seven articles describing the use of herbal medicines after interventional therapy in cardiovascular diseases. There are two clinical research articles describing the efficacy and mechanisms of HM. An article by S.-L. Wang et al. evaluated the 10-year effectiveness of HM plus conventional treatment versus conventional treatment alone with decision-analytic model for ACS after PCI. The authors found that treatment with HM, as an adjunctive therapy, in combination with conventional treatment for 6 months might improve the long-term clinical outcome in ACS patients after PCI. M. Xue et al.' study showed that Xuefuzhuyu oral liquid could effectively improve blood stasis syndrome and aspirin resistance by inhibiting ADP-induced platelet aggregation and patients with the rs5911 genetic variant exhibited better drug response. There are three experimental articles that make in-depth exploration about mechanisms of HM for cardiovascular diseases. Study by J. Su et al. showed luteolin could ameliorate hypertensive vascular remodeling mediated by the regulation of MAPK signaling pathway and the production of ROS. A study by W. Liu et al. demonstrated antihypertensive mechanism of gastrodin involved in regulation of the renin-angiotensin-aldosterone system (RAAS) and PPAR*γ*. F. Wu et al. found that activation of local CSE-H2S-VEGF axis might participate in proangiogenesis effects of Danhong injection, suggesting a potential therapy for diabetic patients with critical limb ischemia. An interesting article by H. Berrougui et al. gives evidence from the beneficial role of extra virgin olive oil (EVOO) consumption towards oxidative stress and cardiovascular diseases. The only meta-analysis as a part of this special issue by A. Liu et al. demonstrated possible efficacy of active compounds of rhubarb root and rhizome that have potential neuroprotective effect for experimental ischemic stroke but should be interpreted with caution because of shortage of the methodology.

From the above-mentioned articles, this special issue provides recent evidence about efficacy, mechanisms, and safety of herbal medicine after interventional therapy in CVDs. We hope this special issue will offer a new scientific understanding of the effect of herbal medicine for CVDs.

*Dazhuo Shi* *Dazhuo Shi* *Michael Y. H. Shen* *Michael Y. H. Shen* *Honglin Luo* *Honglin Luo* *Yan Ma* *Yan Ma*

